# Born from pre-eclamptic pregnancies predisposes infants to altered cortisol metabolism in the first postnatal year

**DOI:** 10.1530/EC-15-0084

**Published:** 2015-09-16

**Authors:** Fiona Broughton Pipkin, Hiten D Mistry, Chandrima Roy, Bernhard Dick, Jason Waugh, Rebecca Chikhi, Lesia O Kurlak, Markus G Mohaupt

**Affiliations:** 1 Department of Obstetrics and Gynaecology, School of Medicine, University of Nottingham, Nottingham, NG5 1PB, UK; 2 Departments of Nephrology, Hypertension, Clinical Pharmacology and Clinical Research, University of Bern, 3010, Berne, Switzerland; 3 Leicester Royal Infirmary, Leicester, LE1 5WW, UK

**Keywords:** steroid hormones, pre-eclampsia, infants, urine

## Abstract

Pre-eclampsia leads to disturbed fetal organ development, including metabolic syndrome, attributed to altered pituitary-adrenal feedback loop. We measured cortisol metabolites in infants born from pre-eclamptic and normotensive women and hypothesised that glucocorticoid exposure would be exaggerated in the former. Twenty-four hour urine was collected from infants at months 3 and 12. Cortisol metabolites and apparent enzyme activities were analysed by gas chromatography-mass spectrometry. From 3 to 12 months, excretion of THS, THF and pregnandiol had risen in both groups; THF also rose in the pre-eclamptic group. No difference was observed with respect to timing of the visit or to hypertensive status for THE or total F metabolites (*P*>0.05). All apparent enzymes activities, except 17α-hydroxylase, were lower in infants at 12 compared to 3 months in the normotensive group. In the pre-eclamptic group, only 11β-HSD activities were lower at 12 months.17α-hydroxylase and 11β-HSD activities of tetrahydro metabolites were higher in the pre-eclamptic group at 3 months (*P*<0.05). 11β-hydroxylase activity increased in the pre-eclamptic group at 12 months. Cortisol excretion, determined by increased 11β-hydroxylase, compensates for high 11β-HSD-dependent cortisol degradation at 3 months and at 12 months counterbalances the reduced cortisol substrate availability in infants born from pre-eclamptic mothers.

## Introduction

The fetal adrenal gland and hypothalamic–pituitary axis (HPA) play important roles during pregnancy. Between weeks 32 and 34 of gestation, there is a rapid maturation of the fetal adrenal cortex, allowing development of a variety of mechanisms associated with a successful transition to extrauterine life (1, 2, 3, 4). Frequently, intrauterine growth restriction (IUGR) and premature delivery are due to pre-eclampsia (5), a pregnancy-specific syndrome occurring in ∼3% of all pregnancies and one of the leading causes of maternal/perinatal mortality and morbidity worldwide (6, 7). Various organ functions in these infants are subject to intrauterine developmental reprogramming (8, 9), with evidence of decreased pituitary adrenal feedback control of glucocorticoid maintenance and an association with metabolic syndrome (10, 11).

Little is known about steroid hormone availability and metabolism during the early *postpartum* period in infants. Fetal exposure to maternal cortisol is low, as it is tightly regulated by the placental enzyme 11β-hydroxysteroid-dehydrogenase type 2 (11β-HSD), assumed to protect the fetus against high active levels of glucocorticoids. Enhanced maternal ‘stress’, reduced placental cortisol inactivation due to mechanisms such as a compromised placental 11β-HSD activity in pre-eclampsia (12) and/or a hypoxic fetal environment, might lead to increased fetal exposure to cortisol in pregnancy and during birth (13, 14, 15). Fetal exposure to exogenous glucocorticoids, to enhance lung maturation, is a further confounder for fetal cortisol exposure (16). The premature infant must counterbalance the hormonal demands of acute critical stress against those of growth and organ maturation (17). Corticotrophin-releasing hormone (CRH) challenge tests in premature infants have demonstrated that the ability of the pituitary to respond adequately to CRH stimulation depends not only on the dose of CRH, but also on the maturity of the pituitary–adrenal axis (18, 19). Two types of response are conceivable: First, adrenal cortical responsiveness could be downregulated at birth due to the relatively high prenatal glucocorticoid exposure. Second, given compromised intrauterine development, the adaptation to later life requirements for steroid hormone production could be enhanced by an already dysfunctional central feedback response. As adrenal and central nervous perfusion is maintained even during fetal distress, any change might be subtle and only apparent when a need for sensitive regulation arises.

**Figure 1:**
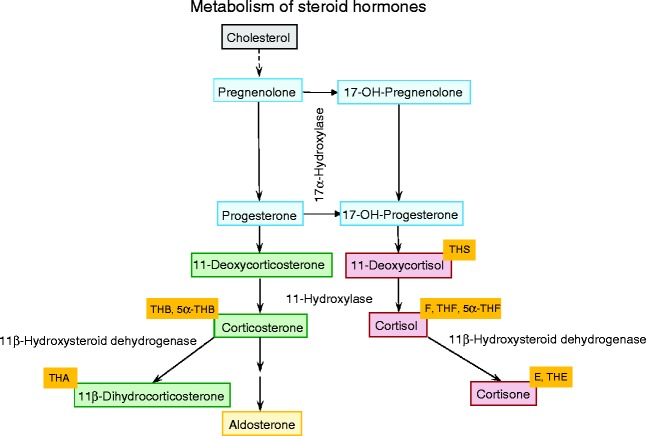
Schematic flow diagram representing the steroid hormone pathways regulating cortisol availability. Progesterone-, corticosterone- and cortisol-related steroid hormones are labelled blue, green and red respectively. Steroid hormone metabolites used to assess a given steroid hormone are shown in yellow boxes. Enzymes, whose apparent activities have been calculated within the manuscript, are marked.

Though potentially biased by the fact that non-surviving fetuses cannot be evaluated, we hypothesised that glucocorticoid exposure is exaggerated after pre-eclampsia. We aimed to investigate cortisol metabolism (Fig. 1), at postnatal months 3 and 12 in infants born to pre-eclamptic and normotensive mothers.

Previous studies have used immunoassays which have the problem of steroid cross reactivity (20, 21). We have addressed this problem by utilising gas chromatography-mass spectrometry (GC-MS) (22). In addition, we have measured steroids in 24 h urine collections, which enables a non-invasive assessment of hormonal production rates and reflects approximately 70% of the cortisol production rate in adults, as determined by stabilised isotope dilution technique (23).

## Material and methods

### Subjects

This study was performed as part of a considerably larger study comparing infants born to pre-eclamptic and suitably matched normotensive pregnancies with the aim of 100 in each group. Although a majority of these women initially agreed to collect nappies, a number of these dropped out at 3 months and even more so at 12 months, leaving final group sizes of normotensive (3 months: *n*=50; 12 months: *n*=29) and pre-eclamptic (3 months: *n*=45; 12 months: *n*=16) pregnancies. Infants of pre-eclamptic mothers were matched to normotensive controls by ±1 week of gestation age at delivery.

In this cross-sectional study, all parents of the participants, who were recruited from Leicester Royal Infirmary Teaching Hospital, gave informed, written consent to participation on behalf of their infants. The protocol was approved by the Trent Multicentre Research Ethics Committee. Infants born from a pre-eclamptic pregnancy and closely matched (see the previous discussion) infants born to normotensive pregnancies were eligible for the study. Pre-eclampsia was stringently defined using the International Society for the Study of Hypertension in Pregnancy guidelines of systolic blood pressure of 140 mm Hg or more and diastolic pressure (Korotkoff V) of 90 mm Hg or more on two occasions after 20 weeks gestation in a previously normotensive woman, together with proteinuria of ≥300 mg/l, ≥500 mg/day or ≥2+ on dipstick analysis of midstream urine (MSU) if 24-h collection result was not possible (24). Infants with low birth weight due to congenital infections such as cytomegalovirus, dysmorphism, twins, severe cerebral palsy, congenital heart disease, acute infections, hyaline membrane disease or other respiratory disease, jaundice, hypoglycaemia or with a history of urinary tract infections, urological or renal problems were excluded. Demographic data for the children were recovered from the hospital records and are presented in Table 1. The corrected birthweight percentile for each infant was computed, correcting for gestational age at delivery, gender, maternal parity, and BMI (25).

### Urinary sampling

Urine was collected at postnatal months 3 and 12 months, over 24 h using pure cellulose disposable nappies provided in advance to the mothers. A thin gauze was placed between the baby's skin and the surface of the inner side of the nappy to reduce contamination with meconium or stool (26); samples with diarrhoea were omitted. Nappies of the appropriate size were changed every 4 h for term and 8 h for preterm infants and were weighed before and after urine collections, allowing exact calculation of 24-h urine output (27).

Nappies were stored in plastic bags at room temperature until completion of the 24-h collection period and then stored at −20 °C. The 24-h urine was extracted using established methods, with recovery of steroid hormones similar to those previously tested (26). To extract urine, nappies were defrosted, folded inside out, and placed in a plastic bag before extraction using a specially constructed hydraulic press. The extracted urine for the 24-h collection was pooled and centrifuged. The collected specimens were stored at −80 °C prior to GC-MS analysis.

### Assessment of urinary steroid hormones by GC-MS

Steroid hormone measurement by GC-MS was performed on a Hewlett-Packard gas chromatograph 6890 (Hewlett-Packard) with mass selective detector 5973 by selective ion monitoring, as described earlier (28). This method enabled the following metabolites to be determined: tetrahydro-deoxycortisol (THS), cortisol (F), cortisone (E), tetrahydro-F (THF), 5α-THF (5αTHF), tetrahydro-E (THE), tetrahydro-corticosterone (THB), 5α-Tetrahydro-corticosterone (5α-THB), TH-dehydro-B (THA) and pregnandiol. Total F metabolites were calculated as the sum of the cortisol and cortisone metabolites. Steroid hormone concentrations are provided as μg/24 h.

By convention, enzyme activities are calculated by ratios of various metabolites (29). The enzyme activities described are therefore apparent activities and not directly measured. The apparent activity of 17α-hydroxylase was calculated as the ratio of (THA+THB+5αTHB)/ (THE+THF+5αTHF) and the apparent 11β-hydroxylase activity as 100xTHS/(THE+THF+5αTHF). For these apparent activities, high ratios represent low enzyme activities and *vice versa*.

11β-hydroxysteroid dehydrogenase exists in two forms: 11β-HSD1 and 11β-HSD2. 11β-HSD1 reduces cortisol to the less active cortisone; the reverse reaction is also possible. Conversely, 11β-HSD2 irreversibly reduces cortisol to cortisone. The net cortisol to cortisone balance in response to a given total 11β-hydroxysteroid dehydrogenase enzymatic activity was assessed by calculation of the ratio of F/E and the corresponding ratio of their TH metabolites (THF+5αTHF)/THE (30).

### Statistical analyses

Data are summarised as mean±s.d. or median (IQR) as appropriate for the distribution. Correlations between gestational age and steroid hormone excretion or enzyme activity were assessed using Spearman's correlation analysis for normotensive and pre-eclamptic groups separately and/or combined. Mann–Whitney *U* test was used to analyse the subgroups, as appropriate according to standard normality testing. Unpaired student's *t*-test was used to compare normotensive and pre-eclamptic groups if standard normal distribution was confirmed. All the statistical analyses were performed using SYSTAT version 11 (SYSTAT Software, Inc., Erkrath, Germany). Significance was assigned at *P*<0.05.

## Results

### Clinical characteristics

We recruited 95 infants at 3 months and 45 infants at 12 months, all from singleton pregnancies. A summary of basic demographic data are given in Table 1. It can be seen that gestation ages were comparable at both time intervals.

### Cortisol metabolism at 3 and 12 months, related to gestational week at delivery in infants born to normotensive or pre-eclamptic mothers

**Figure 2:**
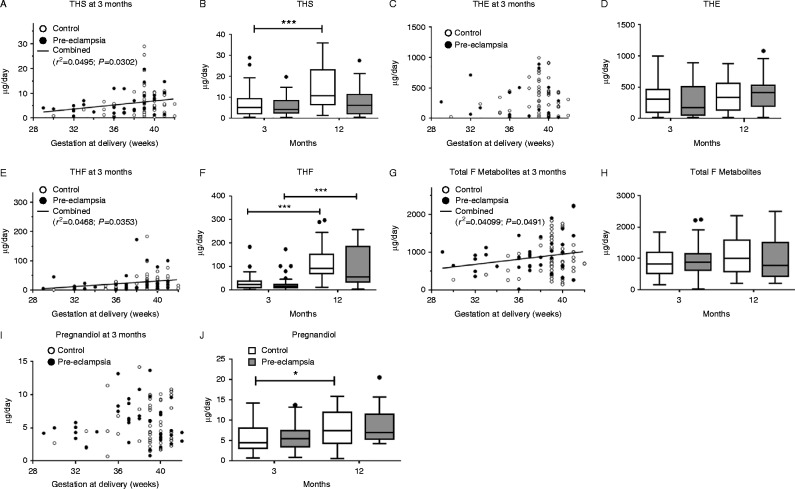
Cortisol metabolites at 3 and 12 months after birth in infants born to normotensive (3 months: *n*=50; 12 months: *n*=29; ○ or white bars) or pre-eclamptic (3 months: *n*=50; 12 months: *n*=16; ● or grey bars) pregnancies, related to gestational week in infants. At 3 months, positive associations were observed for (A) THS (*r*=0.2225, *P*=0.0302), (E) THF (*r*=0.2163, *P*=0.0353) and (G) Total F (*r*=0.2022, *P*=0.0491) in the data overall. Specific metabolites include (A and B) tetrahydro (TH)-metabolite of deoxycortisol (S) (THS). (C and D) tetrahydro-E (THE); (E and F) TH-cortisol (THF); (G and H) Total F metabolites and (I and J) Pregnandiol. Data presented as median (IQR); *P<0.05; ***P<0.001.

As can be seen from Fig. 2, there was still a residual effect of gestational age at delivery in relation to THS, THF and total F metabolites (*P*<0.05), excretion being greater as gestation progressed. This relationship was lost at 12 months (Supplementary Figure 1, see section on supplementary data given at the end of this article). Figure 2 also shows summary data for cortisol and the various metabolites in the normotensive and pre-eclamptic groups. There was no difference in any of the measurements of the adrenal cortisol axis. By the 12 month visit, excretion of THS and THF had risen significantly in the normotensive group (*P*<0.05 for both). THF had also risen in the pre-eclamptic group (*P*<0.05). No difference was observed either with respect to timing of the visit or with respect to hypertensive status for F, THE or total F metabolites (*P*>0.1 for all).

Pregnandiol excretion did not differ significantly at either 3 or 12 months between normotensive and pre-eclamptic groups. Both groups showed a rise in pregnandiol excretion between 3 and 12 months, which was significant only in the normotensive group (*P*<0.05).

### Apparent enzyme activities relevant to cortisol metabolism at 3 and 12 months, related to gestational week in infants born to normotensive or pre-eclamptic pregnancies

**Figure 3:**
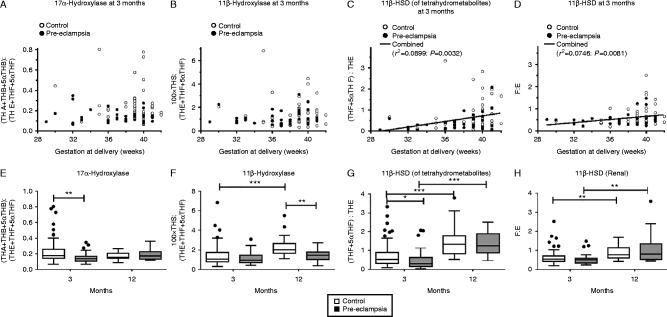
Urine apparent enzyme activities at 3 and 12 months after birth in infants born to normotensive (3 months: *n*=50; 12 months: *n*=29; ○ or white bars) or pre-eclamptic (3 months: *n*=45; 12 months: *n*=16; ● or grey bars) pregnancies, related to gestational weeks in infants. At 3 months, positive associations were observed for both apparent measure of (C) 11-β-HSD, (of tetrahydrometabolites: *r*=0.2998, *P*=0.0032) and (D) (renal: *r*=0.273, *P*=0.0081) in the data overall. Specific metabolites include (A and E) 17α- Hydroxylase (B and F) 11β-Hydroxylase; (C and D) 11-β-HSD (of tetrahydrometabolites) and (D and H) 11-β-HSD (renal). Low ratios indicate high apparent activities, whereas high ratios signify low conversion from substrate to the product. Data presented as median (IQR0); *P<0.05; **P<0.01; ***P<0.001.

Figure 3A displays cortisol substrate delivery, B substrate production, and C and D cortisol degradation.

Figure 3C and D shows that regardless of the putative primary site of cortisol degradation (hepatic or renal), there was still a significant residual effect of gestational age at delivery. This did not differ between normotensive and pre-eclamptic groups. It should be remembered that high ratios represent low activities.

Figure 3A and B shows that at 3 months, gestational age at delivery was not associated with either 17α-hydroxylase or 11β-hydroxylase activity. No association between gestational age and apparent 17α-hydroxylase or 11β-hydroxylase activity was observed at 12 months in infants born either from normotensive or pre-eclamptic pregnancies (Supplementary Figure 1, see section on supplementary data given at the end of this article).

Figure 3E and G show the metabolite ratios, at 3 months, were significantly lower in the pre-eclamptic compared to normontensive controls, indicative of higher 17α-hydroxylase and 11β-HSD activities of tetrahydrometabolites.

The significant increases in the metabolite ratios between 3 and 12 months were indicative of lower activities of all enzymes except 17α-hydroxylase activities in the normotensive group (Fig. 3F, G and H). A similar pattern was only seen in the pre-eclamptic group for the two ratios indicative of 11β-HSD activities (Fig. 3G and H). Thus, there was a significant difference in the 11β-hydroxylase activity between normotensive and pre-eclamptic groups at 12 months, which was not present at 3 months (Fig. 3F).

## Discussion

This is one of the first studies of urinary cortisol metabolites in infants through the first year of life using highly sensitive and specific mass spectrometry-based methodology. It provides data regarding infants exposed to a pre-eclamptic or normotensive pregnancy. In this analysis, the urinary excretion of individual cortisol metabolites and combined cortisol metabolites at 3 months postnatally, still shows an effect of gestational age at delivery in both groups. This highlights the importance of taking into consideration gestational age at delivery in the interpretation of steroid excretion, over at least the first 3 months of postnatal life. The fetal adrenal undergoes a maturational spurt between 32 and 34 weeks (31), considered to be preparing the fetus for birth, as it is followed by the rapid maturation of a variety of other hormone systems. Infants in our study born before this time will not have been exposed to this, which may be associated with compromised adrenal maturation.

The excretion of THS represents substrate availability: in the normotensive group this rises between 3 and 12 months postnatally. THF also rises in parallel to this, although 11β-hydroxylase activity has fallen in this group, the substrate availability more than compensates. THF also rises between 3 and 12 months in the pre-eclamptic group, despite both substrate availability and 11β-hydroxylase activity remaining almost constant over this time. However, the significant fall in both forms of 11β-HSD activity, may have enabled this rise in THF. The increased apparent 11β-hydroxylase activity in the pre-eclamptic group by comparison to the normotensive group at 12 months is in line with the observed maintained F, THF and total F excretion, despite the lower THS excretion. The lack of increase over time of pregnandiol in the pre-eclamptic group implies a lower substrate delivery.

In normotensive pregnancies, the increase in precursors and production of cortisol is to be expected as the adrenal cortex develops from the fetal to the definitive fasciculate zone (18) as can be seen Fig. 2 in response to gestational age at delivery.

Reduced placental 11β-HSD activity, which has been described before (32) does not translate into reduced systemic apparent 11β-HSD activity during the first year of life. Whereas we identified significant falls in both forms of 11β-HSD activity in infants of both normotensive and pre-eclamptic pregnancies during the first postnatal year, Planck *et al*. investigating SGA infants aged between 2 and 13 years, could no longer identify major changes in enzyme activities (33). Likewise, our previous data on high placental cortisol availability in preterm gestation (34) are not reflected in the cortisol production during the first 12 months after birth, but could be a stimulus for the high fetal 11β-HSD activity, which we found at 3 months. Of interest, the reduced ACTH-induced stimulation of cortisol release in preterm infants (35) was not reflected by baseline cortisol production in our study.

Despite a rich literature on HPA maturation in conditions such as intrauterine hypoxia with an excess of cortisol (36), little information is available concerning cortisol metabolites in the infants from pre-eclamptic mothers. Our findings suggest that the steroid hormone metabolism of preeclampsia does not reflect the profile seen in hypoxia. The compromised upregulation of the cortisol precursor, deoxycortisol could be due to the availability of the major steroid hormone precursor cholesterol, or its transport, might be affected due to pre-eclamptic intrauterine conditions and placental dysfunction. This assumption is supported by a smaller increase in pregnandiol in the pre-eclamptic group. Though the data in early childhood are not available, data at birth indicate reduced delivery of cholesterol to the fetus in IUGR and pre-eclampsia (37, 38, 39). The potentially compromised cholesterol transport due to fetal programming may potentially contribute to later life vascular disease and atherosclerosis in these infants (40).

As previously mentioned, by 3 months there was a substantial dropout in attendance at the research unit, which was exacerbated by 12 months. One of us (CR), attempted to contact women at their homes, but there were numerous logistical issues; part of these related to collection of nappies. An additional limitation lay in the use of pure cellulose nappies because of their reduced capacity to absorb urine, thus complete 24-h collection may not have been achieved. Incomplete voiding in these very young infants might have led to limited comprehensive or incomplete urine sampling. This limitation only affects total steroid hormone amounts and not the ratios derived. As muscle mass will be different in preterm and term infants (41) and stability of creatinine was not tested thoroughly for the nappies used at initial study entry, we only provide absolute daily excretion of steroid hormones. We could not control for a limited nappy volume, which might be overwhelmed by strong voiding.

In conclusion, in infants born from pre-eclamptic mothers, cortisol excretion is determined by an increase in 11β-hydroxylase, which compensates for the high 11β-HSD-dependent cortisol degradation at 3 months. This might still be present, after protecting the fetus from high placental cortisol leakage during pre-eclamptic conditions (34). The elevated apparent 11β-hydroxylase at 12 months appears to counterbalance the reduced substrate availability resulting in an unaltered total cortisol metabolite excretion. Future research should be directed to understand the role and development of salvage mechanisms, such as reverse cholesterol transport by enzymes such as the 27α-hydroxylase.

## Supplementary data

This is linked to the online version of the paper at http://dx.doi.org/10.1530/EC-15-0084.

## Author contribution statement

H D Mistry, C Roy, L O Kurlak, B Dick and R Chikhi performed the experiments and analyses and drafted the paper. F Broughton Pipkin and M G Mohaupt initiated and guided the research and corrected drafts of the paper. J Waugh reviewed the research and analyses and reviewed the writing of the paper.

## Figures and Tables

**Table 1:** Pregnancy and infant demographic and outcome data for these unpaired cross-sectional samples at 3 and 12 months^a^.

	**Normotensive controls**	**Pre-eclampsia (PE)**
3 months (*n*)	50	45
No. of early-onset PE (≤34 weeks)		12
No. of late-onset PE (>34 weeks)		33
Max systolic BP (mmHg), median (min, max)		157 (132, 211)
Max diastolic BP (mmHg), median (min, max)		110 (91, 132)
Gestational age at delivery (weeks)	38.8±2.3	37.0±3.1
Post conception age (weeks)	50.7±2.2	50.2±1.9
Birthweight (g)	3291±584	2730±895*
Corrected birthweight centile	46 (25, 74)	28 (2, 55)*
SGA, *n* (%)	6 (12)	20 (44)
Gender (male *n* (%))	29 (58)	22 (49)
24 h urine volume (ml)	602.8±189	611.6±192.6
Preterm (*n* (%))	9 (18)	14 (31)
Infant weights at 3 months (g)	6180±1023	5474±1243*
12 months (*n*)	29	16
No. of early-onset PE (≤34 weeks)		4
No. of late-onset PE (>34 weeks)		12
Max systolic BP (mmHg), median (min, max)		161 (145, 187)
Max diastolic BP (mmHg), median (min, max)		110 (98, 122)
Gestational age at delivery (weeks)	38.6±2.7	37.6±2.2
Post conception age (weeks)	90.6±2.7	89.5±2.1
Birthweight (g)	3243±701	2952±850
Corrected birthweight centile	32 (12, 68)	27 (1, 54)
SGA, *n* (%)	7 (24)	7 (44)
Gender (male *n* (%))	18 (62)	7 (44)
24 h urine volume (ml)	570±225.7	704.7±330.8
Preterm (*n* (%))	6 (21)	6 (38)
Infant weights at 12 months (g)	9785±1322	9420±1709

*P<0.05.

a Data presented as either mean±s.d. or median (IQR).
